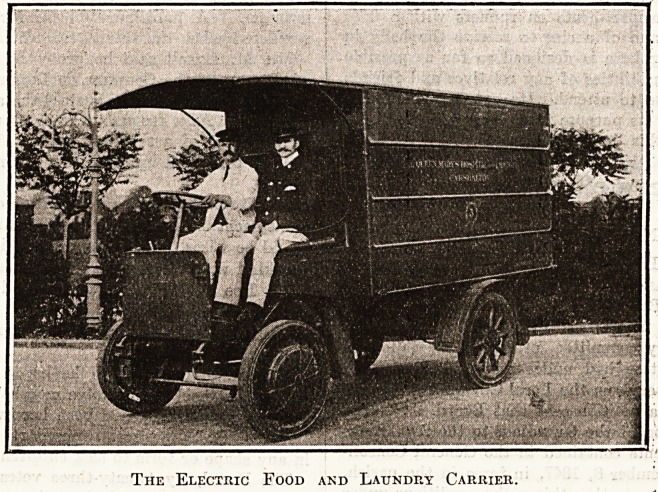# Electric Motors as Food Carriers

**Published:** 1913-11-22

**Authors:** 


### PRACTICAL POINTS.

**(Criticism and Suggestions Invited.)**

### Electric Motors as Food Carriers.

### THE VEHICLES IN USE AT QUEEN MARY'S HOSPITAL, CARSHALTON.

It is only where the cottage-block system is in use that the need for electric motors as food carriers is of immediate importance, perhaps, to the institutional world. But as a system involving them is in daily use at Queen Mary's Hospital, Carshalton, a detailed description of the vehicles becomes of interest.

The electric motor vehicle used for carrying food, milk, laundry baskets, etc., to and from the wards, which are in the form of cottage blocks, at Queen Mary's Hospital for Children, Carshalton, is of special interest from the fact that a central kitchen has rarely so much ground to cover as at this institution.

The van has a carrying capacity of 30 cwt. The weight of the body is about 7 cwt.; the wheel base is 8 feet 6 inches; the track 5 feet; and the inside dimensions 9 feet by 4 feet 9 inches by 5 feet high, the height of the roof from the ground being 8 feet 9 inches. The weight of the chassis, including the two patent wheel motors, is 33 cwt., and the battery weighs 12 cwt.

The Electric Food and Laundry Carrier.

The vehicle is front-driven, the two front wheels being powerful independent electric motors, an arrangement which does away with all transmission gear. Each of the motors can develop 20 e.h.p. if required; they are dust and water tight. The battery, which consists of forty-four Tudor Company's Ky.C.9 cells (195 ampère hours), is carried under the driver's seat, and is of sufficient capacity to drive the vehicle at a speed of about twelve miles an hour, and to give it a radius of about forty miles with one charge. It is connected up to a controller which allows of five different speeds ahead and two reverse speeds with an electro-magnetic brake. There is an auto-magnetic switch, connected to the controller lever and foot pedal, which makes it impossible to change speeds while the motors are in circuit. This does away with the possibility of an unskilled driver injuring the motors, and the vehicle can be driven by a porter after only a few days' tuition.

At Queen Mary's Hospital the continuous current required to charge the battery is obtained by transforming the alternating current by means of a motor generator. The average time taken each night to charge the battery after a twelve-mile run is five hours. This charging is usually watched by one of the night staff, but once a week the electrical fitter examines the cells, takes the specific gravity and temperature, and supervises the charging.

The cost of the vehicle was £499 10s. The expenses of running for one year amounted to £249 12s. 8d.:—Current for charging battery, £30 11s. 9d.; battery company's charges, £27 12s.; acid and distilled water, £3; services of engineering staff, valued at £16 19s. 7d.; tyres, £25; services of two porters, valued at £146 9s. 4d.

The vehicle was supplied by the "Cedes" Electric Traction, Limited, of 112 Great Portland Street, W.

## Figures and Tables

**Figure f1:**